# Examining the relationship between perceived teacher support and students’ academic engagement in foreign language learning: Enjoyment and boredom as mediators

**DOI:** 10.3389/fpsyg.2022.987554

**Published:** 2022-09-20

**Authors:** Yanlin Zhao, Lianrui Yang

**Affiliations:** College of Foreign Languages, Ocean University of China, Qingdao, China

**Keywords:** perceived teacher support, enjoyment, boredom, academic engagement, second language learning

## Abstract

As an important contextual factor influencing various aspects of students’ learning, teacher support has been widely explored in general education but largely overlooked in the English as a Foreign Language (EFL) context. Given its potential positive role in students’ academic performance, the present study intended to investigate the relationship between perceived teacher support, enjoyment, boredom, and academic engagement in the EFL context. In so doing, 1094 Chinese high school students were recruited to complete the online questionnaire of the four variables. SPSS and PROCESS macro were used for descriptive, correlational, and mediation analyses. The results showed that students had moderate levels of perceived teacher support, enjoyment, and academic engagement and a low level of boredom. Further correlation analyses indicated medium to large correlations between perceived teacher support, enjoyment, boredom, and academic engagement. Enjoyment and boredom collectively mediated the relationship between perceived teacher support and academic engagement. Directions for future research and implications for researchers and educators are presented at the end.

## Introduction

The advancement of Positive Psychology in Second Language Acquisition (SLA), which aims to help students to flourish (MacIntyre, 2016), has established the critical roles of teacher-related factors, positive and negative emotions, and academic engagement in students’ language learning (e.g., Shao and Parkinson, 2021; Derakhshan et al., 2022a; Li, 2022). Teacher-related factors have been widely studied because teachers play important roles in influencing students’ psychological wellbeing and language learning achievement (Mercer et al., 2018). Among teacher-related factors, teacher support as a combination of various types of support that teachers offer to their students has the potential to foster a positive relationship between teachers and their students (Lei et al., 2018). Previous studies in general education indicated that it could positively influence students’ psychological states and learning behaviors (e.g., Hao et al., 2017; Ma et al., 2021; Sadoughi and Hejazi, 2021). However, the influence of teacher support has only received scant attention in the English as a Foreign Language (EFL) context (Sadoughi and Hejazi, 2021). Given its role as an important source of students’ language skill development and emotional experience (Piechurska-Kuciel, 2011; De Ruiter et al., 2019), teacher support should be further explored.

As the most frequently experienced emotions in the EFL context, students’ enjoyment and boredom can significantly influence students’ learning outcomes (Derakhshan et al., 2022c; Dewaele and Li, 2022). A high level of enjoyment can increase students’ momentary thought-action repertoires (Fredrickson, 2006) and exert a positive influence on students’ academic engagement and language achievement (Dewaele and Li, 2021; Wang et al., 2022). In contrast, a high level of boredom is likely to cause dissatisfaction, inattention, and amotivation (Kruk and Zawodniak, 2018), thus having a deleterious effect on students’ language learning (Derakhshan et al., 2022b). Since the two emotions were introduced into the EFL context, there has been an upsurge of interest in investigating the antecedents and outcomes of the two achievement emotions separately to understand their sources and influence (e.g., Botes et al., 2021; Dewaele and Li, 2022; Kruk et al., 2022b). However, relatively limited attention has been paid to investigating the antecedents and outcomes of the two achievement emotions within the same study to gain a more comprehensive understanding of these emotions (Dewaele and Li, 2021). Such endeavors should be valued in that they cannot only take the mediating roles of emotions into consideration but also provide valuable insights into how language learners’ achievement emotions that influence various aspects of language learning can be regulated by both educators and learners (Shao et al., 2019, 2020a).

As the key concern of all instructed language learning (Dörnyei and Kormos, 2000), students’ academic engagement is another variable under investigation in the present study. Although academic engagement is viewed as a “new kid on the block” (Reschly and Christenson, 2012, p. 4), because of its close linkage with students’ learning actions and academic success, it has experienced an exponential increase in the last 20 years (Hiver et al., 2021; Zhou et al., 2021). As the outcome of the interaction between both individual factors and the instructional context (Baker et al., 2008), academic engagement is not only influenced by teacher-related factors such as teacher support and enthusiasm (Dewaele and Li, 2021; Hejazi and Sadoughi, 2022) but also affected by students’ emotional and motivational variables (Mystkowska-Wiertelak, 2020; Dewaele and Li, 2021). However, only sporadic studies have explored the relationship between engagement and students’ emotional variables and these limited studies indicated that positive emotions like enjoyment could promote students’ academic engagement (e.g., Guo, 2021; Liu, 2022), while negative emotions like boredom could make students disentangle from classroom learning activities (e.g., Dewaele and Li, 2021; Derakhshan et al., 2022b). Further exploration is needed to better understand the influence of students’ achievement emotions on their engagement (Derakhshan et al., 2022b).

Under the guidance of the control-value theory, which emphasizes the mediating role of achievement emotions in the relationship between social environments and students’ academic performance (Shao et al., 2019), the present study aimed to investigate how enjoyment and boredom mediate the influence of perceived teacher support on students’ academic engagement. As above-mentioned, this study differs from previous ones in the following three aspects. First, this study explores an important but largely ignored teacher-related factor, i.e., teacher support, as an antecedent. Second, the antecedent and outcome of the two emotions are explored together, which offers a more comprehensive understanding when compared with previous studies that only focus on one aspect. Third, engagement is explored as an outcome of enjoyment and boredom, which lacks clarity in previous studies.

## Literature review

### Emotion research in SLA

The introduction of Positive Psychology in SLA makes researchers look beyond cognitive factors and pay close attention to positive psychological factors (Shao et al., 2020a; Dewaele and Li, 2022). A bunch of positive emotions and personal traits is embraced into the field of foreign language learning (MacIntyre, 2016), among which enjoyment and engagement have received a lot of scholarly attention because of their benefits for students’ language learning achievement (Wang et al., 2021). However, what needs to be warned is that Positive Psychology does not mean that negative emotions can be overlooked and margined (Shao et al., 2020a; Wang et al., 2021). Boredom, as the most pervasively experienced emotion that may cause dissatisfaction, amotivation, and attention deficit (Pawlak et al., 2021), has witnessed a growing number of studies conducted in Polish, Chinese, and Iranian EFL contexts (e.g., Kruk, 2016; Derakhshan et al., 2021; Dewaele and Li, 2021; Zhang L. J. et al., 2022). Moreover, along with the recognition of the importance of emotions in language learning, investigation into its influential factors has also received increased attention (e.g., Shao et al., 2013; Derakhshan et al., 2022b; Zhang L. J. et al., 2022). Due to the fact that language learning requires a lot of interaction between teachers and students (Ellis, 2015), the influence of teacher-related factors is highly valued (e.g., Li, 2022; Wang et al., 2022).

With a growing number of studies separately exploring the antecedents and outcomes of students’ achievement emotions, researchers (e.g., Shao et al., 2020b; Li, 2021) have borrowed the control-value theory of achievement emotions from general educational psychology and used it as a theoretical framework to build a relationship between achievement emotions and its antecedents and outcomes. The application of this theory not only draws researchers’ attention to the investigation of the antecedents and outcomes of achievement emotions within the same study but also promotes a comprehensive understanding of students’ achievement emotions through integrating various theoretical propositions (e.g., Shao et al., 2019, 2020a; Dewaele and Li, 2021). In the present study, this theoretical framework was used to link enjoyment and boredom to perceived teacher support (i.e., the antecedent) and academic engagement (i.e., the outcome).

### Teacher support

Teacher support can be defined as including teachers’ caring, understanding, dedication, dependability, and friendliness toward their students (Ryan and Patrick, 2001). It is an important component of social support in the classroom context (Tardy, 1985). Supportive teachers value and are interested in establishing personal relationships with their students, and they can provide help, assistance, and advice to their students in need (Ryan and Patrick, 2001; Liu et al., 2021). Timely help from the teacher is likely to make students feel safe and motivated (Wentzel et al., 2017), encourage them to put more effort into the learning process, become more engaged in learning activities and achieve better learning outcomes (Mercer et al., 2011; Ma et al., 2021).

As a multifaceted construct, teacher support has been defined differently from different theoretical perspectives. According to the self-determination perspective, teacher support incorporates three dimensions, i.e., support for autonomy, involvement, and structure (Lei et al., 2018). From a social support perspective, teacher support can be defined as a teacher providing informational, instrumental, appraisal, and emotional support to their students (Malecki and Demary, 2002). Informational support refers to providing students with information or advice on a particular subject area; instrumental support is offering time, service, or skills to students; appraisal support is giving students evaluative feedback; emotional support means providing students with love, trust, and empathy (Malecki and Elliott, 1999).

As an important contextual factor, teacher support is a valuable resource for students to increase their academic engagement, improve learning achievement, and cultivate positive emotions toward the learning process (e.g., Suldo et al., 2009; Strati et al., 2017). Conner and Pope (2013) found that teacher support was significantly positively related to students’ behavioral, cognitive, and emotional engagement. In addition, teacher support can also indirectly influence students’ academic engagement through increasing their positive achievement emotions and buffering their negative achievement emotions (Ekatushabe et al., 2021; Sadoughi and Hejazi, 2021). Existing studies on teacher support were mainly conducted in general education (Sadoughi and Hejazi, 2021), while limited attention is paid to its influence on students’ second language learning. As the communicative nature of language learning and frequent interactions between teachers and students in language classes have identified the teacher as an important source of encouragement and support for their students (Jiang and Dewaele, 2019), teacher support, as a core teacher-related factor, should be further explored (Sadoughi and Hejazi, 2021).

### Enjoyment

Enjoyment has received increasing scholarly attention along with the development of positive psychology (e.g., Pan and Zhang, 2021; Shen, 2021; Liu, 2022). Enjoyment is regarded as a component of flow, and it refers to a positive state in which challenges and the skills needed to deal with them are in line with each other (Csikszentmihalyi, 1990). In other words, students are likely to experience enjoyment when they can deal with the challenges that they meet. Enjoyment is also closely related to pleasure but differs from it in that enjoyment incorporates the notion of successfully dealing with challenges (Boudreau et al., 2018).

As for the dimensions of students’ foreign language enjoyment (FLE), a two-factor structure was first put forward by Dewaele and MacIntyre (2016). They identified two factors, i.e., FLE-Social and FLE-Private in an international sample. Then, Dewaele and Dewaele (2017) extracted a three-dimensional structure, including Peer-controlled versus Teacher-controlled positive atmosphere, FLE-Social, and FLE-Private. Later on, in order to better capture Chinese EFL learners’ enjoyment, Li et al. (2018) adapted the original Foreign Language Enjoyment Scale (Dewaele and MacIntyre, 2016) and proposed a new three-dimensional model which includes FLE-Private, FLE-Atmosphere, and FLE-Teacher.

Concerning factors influencing students’ enjoyment, it was found that students’ enjoyment in educational settings was not only related to learner-internal factors such as motivation and language proficiency level but also to learner-external factors such as the teaching content, classroom environment, peer-related factors, and teacher-related factors (e.g., Dewaele and Li, 2021; Shao and Parkinson, 2021; Shirvan et al., 2021). Moreover, previous studies (Jiang and Dewaele, 2019; Moskowitz and Dewaele, 2020) indicated that enjoyment was more likely to be attributed to teacher-related factors than to learner-related factors. The existing body of research on teacher-related factors has confirmed that students’ enjoyment is closely related to teacher enthusiasm (Dewaele and Li, 2021), friendliness (Dewaele et al., 2019), foreign language use (Dewaele and Dewaele, 2017), etc. Despite these important findings, further attention should be given to other teacher-related factors to find out effective ways to promote students’ enjoyment from the teachers’ perspective. Germane to the present study, teacher support, as an important source for students’ language skill development and emotional experience (Piechurska-Kuciel, 2011; Jin and Zhang, 2018; De Ruiter et al., 2019) was explored in the foreign language learning context.

### Boredom

Boredom is a commonly experienced deactivating emotion in academic settings (Kruk, 2021). It refers to a mild, silent, and unpleasant affective state or psychological experience that makes students show an indifferent attitude toward what goes on around them, and it is a combination of disappointment, dissatisfaction, and amotivation (Kruk and Zawodniak, 2018; Zawodniak et al., 2021). Students tend to be bored when they cannot perceive meaning and pleasure from the ongoing activity (Eastwood et al., 2012).

Different researchers have classified boredom into different types to shed light on the complexities of this construct (Pawlak et al., 2021). In accordance with its stability, boredom can be divided into trait boredom and state boredom. Trait boredom refers to a general tendency to perceive surrounding environments as uninteresting (Bench and Lench, 2013), while state boredom is a context-dependent, transient, and reversible negative feeling (Pawlak et al., 2020). According to the control-value theory of achievement emotions, boredom can be divided along a three-dimensional taxonomy, i.e., object focus (activity vs. outcome), value (positive vs. negative), and activation (activated vs. deactivated) (Pekrun and Stephens, 2010). In line with this division, boredom refers to a negative deactivating emotion that pertains to ongoing achievement-related activities.

Although boredom is a frequently experienced emotion in educational settings, because of its silent and elusive characteristic, it has been undervalued for years in the language learning context (Kruk, 2016). Then, influenced by the affective turn (Pavlenko, 2013), researchers began to explore the sources and solutions of students’ boredom in the EFL context (e.g., Zawodniak et al., 2021; Kruk et al., 2022a). Qualitative studies conducted in diversified English language classes have identified various sources of students’ boredom, and they can be generally divided into three types, i.e., teacher-induced, student-induced, and content-induced boredom (Derakhshan et al., 2021; Zawodniak et al., 2021; Kruk et al., 2022a). As for the possible ways of regulating this deleterious emotion, teachers are regarded as playing a central role in relieving students’ boredom, while students are seen as either escaping from boredom or being overwhelmed by it (Pawlak et al., 2021).

However, up to now, there is a paucity of empirical research using large-scale quantitative designs to investigate the relationship between boredom and other individual and contextual variables (Li, 2022). Some existing studies (e.g., Derakhshan et al., 2022b; Zhang L. J. et al., 2022) indicated that boredom was negatively related to students’ willingness to communicate, academic engagement, and English language achievement. Other researchers focused on the influence of teacher-related factors on students’ boredom and identified the importance of teacher enthusiasm, emotioncy, and friendliness (e.g., Dewaele and Li, 2021). Among these teacher-related factors, teacher support has rarely been studied in quantitative studies in the EFL context. As it has been identified as an important influencing factor for students’ negative achievement emotions in other educational contexts (Lei et al., 2018), further investigation of this relationship in the EFL context is meaningful for finding out possible ways of mitigating students’ boredom.

### Academic engagement

Academic engagement is the core indicator of meaningful learning, and it refers to students’ active involvement and participation in a learning activity (Reeve, 2012; Zhou et al., 2021). Although it is closely intertwined with motivation, the biggest difference between the two concepts is that motivation is intention-oriented, while engagement is action-based (Reschly and Christenson, 2012; Mercer, 2022). Specifically, as a predictor of students’ positive functioning and highly valued learning outcomes, it embraces the notion of action and is context-dependent, dynamic, and malleable in nature (Hiver et al., 2021).

Concerning the dimensions of academic engagement, following the practice of general educational psychology, academic engagement in L2 learning is generally viewed as a multiple-dimensional model encompassing emotional, cognitive, behavioral, and/or social dimensions (Philp and Duchesne, 2016). The most commonly recognized model conceptualizes engagement as encompassing three aspects, that is, behavioral, cognitive, and emotional engagement (Fredricks, 2011). However, the tripartite framework of engagement is challenged for its deficit in comprehensively capturing the complex nature of students’ academic engagement. As argued by Reeve and Tseng (2011), the triple model overlooked students’ agentic involvement, and then agentic engagement was added to indicate students’ constructional, proactive, and intentional contribution to their learning. The proposed four-dimension model is empirically validated in the EFL context (e.g., Eren and Rakıcıoğlu-Söylemez, 2020; Guo, 2021). Based on the assumption that the true potential of students’ engagement cannot be captured by a single dimension (Zhou et al., 2021), engagement in the present study was defined as a multidimensional concept, incorporating behavioral, cognitive, emotional as well as agentic aspects (Reeve, 2012).

To date, scant attention has been paid to the predictors of engagement in L2 learning (Khajavy, 2021), especially the influence of learner-external factors (Oga-Baldwin, 2019). Moreover, although previous studies (e.g., Mercer, 2019; Henry and Thorsen, 2020) have already identified an emotional component in engagement, few studies have been done to explore the relationship between engagement and emotions (Dewaele and Li, 2021). Since engagement is context-specific (Reschly and Christenson, 2012), it is significant to investigate factors influencing students’ language engagement in different contexts to better understand its interaction with contextual variables and students’ personal factors and find out effective ways to get students involved. Given the gaps in the existing literature, the present study sought to explore its relationship with both contextual factors and students’ enjoyment and boredom in the foreign language learning context.

## Research questions

Based on the literature, the following links have been identified: (1) Perceived teacher support is correlated with students’ enjoyment and boredom; (2) Enjoyment and boredom are also linked to academic engagement; (3) Teacher support is related to academic engagement. Correspondingly, a mediating model is proposed, in which boredom and enjoyment collaboratively mediate the effect of perceived teacher support on academic engagement. Specifically, the present study intended to answer the following three research questions:

RQ 1: What are the general tendencies of students’ perceived teacher support, language learning enjoyment, boredom, and academic engagement?

RQ 2: What are the relationships between foreign language learning enjoyment, boredom, perceived teacher support, and academic engagement?

RQ 3: Do foreign language learning enjoyment and boredom mediate the relationship between perceived teacher support and students’ academic engagement?

## Methods

### Participants

A convenience sampling strategy was adopted in the present study. A total of 1181 first-year high school students in 21 classes from three different high schools participated in the present study. However, the data of 87 students was deleted due to short answer time and failure in the lie detector, thus the final sample was 1094 consisting of 499 (45.61%) male participants and 595 (54.39%) female participants. The participants’ age ranged from 14 to 16, with a mean age of 15.44 (*SD* = 0.503). Among them, 361 (33.00%) were in a regular high school (school A), 457 (41.77%) were in a city key high school (school B), and 276 (25.23%) were in a provincial key high school (school C) (see Table 1). The same curriculum regulated by the Ministry of Education of China was followed, and it is aimed to prepare students for the College Entrance Examination held at the end of the third academic year. All the participants were native Chinese speakers and did not have overseas experiences. At the time of data collection, they had eight English classes every week, and each of them lasted for 45 min. Before the data collection, students were informed of the purposes of this study and could choose to participate or not according to their own will. Meanwhile, they were assured that their responses would remain strictly confidential and only be used for research purposes.

**TABLE 1:** Participants’ demographic information (*N* = 1094).

School	No.	Male	Female	Mean age (SD)
A	361	171	190	15.42 (0.50)
B	457	206	251	15.44 (0.50)
C	276	122	154	15.43 (0.50)
Total	1094	499	595	15.44 (0.50)

### Instruments

The questionnaire began with the demographic part which aimed to collect information on students’ age, gender, and overseas learning experience. Following this, four scales aimed at measuring students’ perceived teacher support, enjoyment, boredom, and academic engagement in foreign language learning were presented. In order to eliminate the influence of order effect, survey items were administered in a random manner (Sudina, 2021). These scales were translated into Chinese by the first author, then in order to assure that the translated questionnaire was comprehensible, two Ph.D. students in Applied Linguistics and a professional applied linguist were invited to evaluate the translated items. They were first informed of the research objective and design and then asked to score the acceptability of each item on a 10-point scale. Following the practice of Dewaele and Li (2021), items that were rated below six points were discussed and revised to make them better at eliciting students’ answers. Detailed information on the four scales is presented as follows:

#### Perceived teacher support

Perceived teacher support was measured through the 12-item teacher subscale of the Child and Adolescent Social Support Scale developed by Malecki and Demary (2002). It is based on Tardy’s (1985) social support model and views teacher support as an important source of social support. The teacher subscale divides support from the teacher into four perspectives, i.e., emotional, instrumental, appraisal, and informational support. The teacher subscale is a five-point Likert scale, ranging from *strongly disagree* (1) to *strongly agree* (5), and it has demonstrated sound psychometric properties in previous studies (e.g., Tennant et al., 2015; Yasemin et al., 2019). Items of this subscale were reworded to make it suitable for eliciting students’ perceived support from their English language teachers. An example item is “My English teacher cares about me.” In the present study, the internal consistency was adequate for the overall scale (α = 0.94) and its subscales of emotional (α = 0.87), instrumental (α = 0.82), appraisal (α = 0.76), and informational support (α = 0.80). The construct validity was acceptable (*χ^2^*/*df* = 4.57, CFI = 0.982, TLI = 0.974, SRMR = 0.023, RMSEA = 0.057).

#### Foreign language enjoyment

Students’ enjoyment in English language learning was measured via the Chinese version of the FLE Scale. With a five-point Likert scale ranging from strongly disagree (1) to *strongly agree* (5), this questionnaire contains 11 items and three dimensions, i.e., FLE-Private, FLE-Atmosphere, and FLE-Teacher. Adapted from the original FLE scale (Dewaele and MacIntyre, 2016), it is more appropriate for the Chinese foreign language learning context (Li et al., 2018). In the present study, reliability analysis revealed high internal consistency of the total scale (α = 0.91) and its subscales of FLE-Private (α = 0.87), FLE-Atmosphere (α = 0.82), and FLE-Teacher (α = 0.81). The construct validity was acceptable (*χ^2^*/*df* = 3.43, CFI = 0.986, TLI = 0.980, SRMR = 0.026, RMSEA = 0.047).

#### Boredom

Students’ language learning boredom was assessed using a three-item Likert scale varying from *strongly disagree* (1) to *strongly agree* (5). This scale was adapted by Li (2021) from the Academic Boredom Subscale (Goetz and Hall, 2014) and the Achievement Emotions Questionnaire (Pekrun et al., 2011) to measure students’ English language learning boredom. An example item is “The English learning material bores me to death.” It has demonstrated good internal consistency and validity in empirical studies conducted in the Chinese foreign language learning context (e.g., Dewaele and Li, 2021; Li, 2021). In the present study, the scale showed a high internal consistency (Cronbach’s alpha = 0.82).

#### Academic engagement

Students’ academic engagement was measured through the 22-item Academic Engagement Scale developed by Reeve and Tseng (2011). Responses were rated on a five-point Likert scale ranging from *strongly disagree* (1) to *strongly agree* (5). In this scale, students’ academic engagement is divided into four aspects, i.e., cognitive (eight items; e.g., Before I begin to study English, I think about what I want to get done), emotional (four items; e.g., English class is fun), behavioral (five items; e.g., I listen carefully in English class), and agentic (five items; e.g., I offer suggestions to my English teacher about how to make the class better). This scale was chosen for its comprehensiveness, good construct validity, and high internal consistency. In the present study, Cronbach’s alpha reliability coefficients were 0.84 for the behavioral engagement subscale, 0.81 for the emotional engagement subscale, 0.89 for the cognitive engagement subscale, and 0.85 for the agentic engagement scale, indicating high internal consistency. The construct validity was acceptable (*χ^2^*/*df* = 4.90, CFI = 0.972, TLI = 0.967, SRMR = 0.023, RMSEA = 0.060).

### Procedure and data analysis

The data collection began in early April and ended in late April 2022. First, we contacted five teachers in five different schools and asked them to help with the data collection. Three of them agreed to help and assisted us in getting consent from their school directors and contacting other teachers in their schools. Finally, a total of 11 teachers agreed to be volunteer helpers, and they helped us explain the objective of the present study to their students and sent them the link to the online questionnaire. Finally, the questionnaire was distributed to 21 classes (*N* = 1294) with a response rate of 91.3%. Students who agreed to participate in the study scanned the quick response code and finished the questionnaire in their spare time. In order to show our thankfulness to these participants, a link to some prestored useful learning materials was presented at the end of the questionnaire. If participants need these learning materials, they can download them for free.

After data were collected, they were input into SPSS 24 for processing. Data screening was conducted at first. Since the computer system required students to choose from 1 to 5 for each item, the process of analyzing and missing data was left out. During the data cleansing procedure, we mainly checked and deleted cases that failed in the lie detector (i.e., I am a high school student now). The total number of delated cases was 87, taking up 7.4% of the whole data. After data screening, descriptive analyses and normal distribution tests were conducted. The criteria for normal distribution followed that proposed by Field (2009), according to which data with standardized skewness values between 0 and ±3.0 and standardized kurtosis below ±8.0 was assumed as normally distributed. Next, reliability and validity tests were conducted using SPSS and Amos, respectively. Then, correlational and regression analyses were done using SPSS 24. In the end, mediation analysis was performed using PROCESS v3.3. PROCESS macro was chosen as the analytical tool for the following reasons. First, being introduced by Hayes in 2013, PROCESS macro is now widely used in various fields (Hayes et al., 2017), and it is recognized as one of the major analytical tools for mediation analysis along with Mplus and R (Lemardelet and Caron, 2022). Second, PROCESS requires less effort and programming skills than the SEM program. It makes the mediation analysis simplified, and we can have most of the statistics for interpretation via this macro easily (Igartua and Hayes, 2021).

## Results

To answer the first research question, descriptive analyses were performed using SPSS to explore the general tendencies of students’ perceived teacher support, enjoyment, boredom, and academic engagement. A summary of the mean, range, standard deviations, median, and mode for each variable is presented in Table 2. Based on previous research (Shao et al., 2013; Li, 2019), the total scores of the four variables were divided into three levels, i.e., low, moderate, and high. Based on this standard, varying from 12 to 60, the total score of perceived teacher support below 36 represented a low level, between 36 and 48 was regarded as a moderate level, while 48 and above was a high level. Boredom ranged from 3 to 15, where < 9 indicated a low level, 9–12 a moderate level, and >12 a high level. Within the range of 11 to 55 for enjoyment, <33 indicated a low level, 33–44 a medium level, and >44 a high level. Ranging from 22 to 110 for academic engagement, <66 represented a low level, 66–88 a medium level, while > 88 a high level.

**TABLE 2:** Descriptive statistics for the four variables (*N* = 1094).

Variable	Mean	*SD*	Mdn	Mode	Skewness	Kurtosis
Perceived teacher support	48.84	7.12	48	48	–0.884 (0.074)	3.301 (0.15)
Enjoyment	40.25	8.86	41	44	–0.865(0.074)	1.308(0.15)
Boredom	7.86	2.89	8	6	0.178 (0.074)	–0.563 (0.15)
Academic Engagement	76.05	20.03	79	110	–0.563 (0.074)	0.164 (0.15)

Mdn, the middle value; Mode, the most frequently occurred value.

As can be seen from Table 2, the mean, median, and mode of students’ perceived teacher support were 48.84, 48, and 48, respectively. Further frequency analysis found that 25 (2.29%), 579 (52.93%), and 490 (44.79%) of the 1094 participants reported low, moderate, and high levels of teacher support. This indicated that most of the students perceived a moderate level of teacher support in their English classes. Concerning boredom, the mean, median, and mode were 7.86, 8, and 6, respectively. Additional frequency analysis on boredom found that 629 (57.50%), 413(37.75%), and 52(4.75%) of the participants reported a low, moderate, and higher level of boredom, indicating that most participants had a low level of boredom. As for students’ enjoyment, the mean, median, and mode were 40.25, 41, and 44, respectively. All the three parameters fell into the range of 33–44. Frequency analysis signified that 140 (12.80%), 659 (60.24%), and 295 (26.97%) of them had low, moderate, and high levels of enjoyment, indicating that most of the students had a moderate level of enjoyment in their language learning. In terms of academic engagement, its mean, median, and mode were 76.05, 79, and 110, respectively. A further frequency analysis showed that around 594 (54.3%) of the participants had a medium level of academic engagement, indicating that most of the students were engaged in their language learning.

Concerning the second research question, Pearson correlation analyses were conducted to explore the relationships among the four variables. The results showed that the four variables under investigation significantly correlated with each other (see Table 3). The interpretation of effect size for correlations followed the criteria proposed by Plonsky and Oswald (2014). Specifically, 0.25 is regarded as a small effect size, 0.40 as a medium effect size, and 0.60 as a large effect size.

**TABLE 3:** Correlations among teacher support, enjoyment, boredom, academic engagement (*N* = 1094).

Variable	1	2	3	4
Perceived teacher support	_			
Enjoyment	0.613**	_		
Boredom	–0.424**	–0.460**	_	
Academic engagement	0.491**	0.528**	–0.386**	_

**Indicates statistical significance at a p < 0.01.

Perceived teacher support was found to be significantly correlated with enjoyment, boredom, and academic engagement. Specifically, students’ perceived teacher support was positively related to enjoyment (*r* = 0.613, *p* < 0.01) with a large effect size and negatively linked to boredom (*r* = –0.424, *p* < 0.01) with a medium effect size. In addition, it was also positively related to academic engagement (*r* = 0.491, *p* < 0.01) with a medium effect size. The findings indicated that students who perceived higher levels of teacher support tended to enjoy language learning, get bored less often, and engage in language learning.

Enjoyment showed a statistically significant positive correlation with academic engagement (*r* = 0.528, *p* < 0.01) with a medium effect size, indicating that students with higher levels of enjoyment were more likely to engage in language learning. Additionally, boredom was negatively correlated with enjoyment (*r* = –0.460, *p* < 0.01) and academic engagement (*r* = –0.386, *p* < 0.01) with medium effect sizes. This showed that students who felt less bored were likely to enjoy language learning, show a positive attitude toward the learning process, and be more engaged.

To answer the third research question, a multiple regression analysis was conducted. Before running the regression test, a multicollinearity test was performed at first. As shown in Table 4, all the tolerance values were above 0.1, and all the variance inflation factor (VIF) values were less than 10. According to the criteria put forward by Tabachnick and Fidell (2013), there was no multicollinearity problem between these variables. The results of multiple regressions are presented in Table 4. As the *F* value and degree of freedoms indicated, all the regression equations were significant at *p <* 0.001 level. Students’ perceived teacher support was positively and significantly correlated with their academic engagement (β = 0.491, *p* < 0.001) and enjoyment (β = 0.613, *p* < 0.001). At the same time, it also showed a negative relationship with boredom (β = –0.424, *p* < 0.001). When perceived teacher support, enjoyment and boredom entered the regression analysis together, perceived teacher support still positively predicted students’ academic engagement (β = 0.237, *p* < 0.001) but with a smaller effect size. Enjoyment positively predicted academic engagement (β = 0.318, *p* < 0.001), while boredom negatively predicted academic engagement (β = –0.139, *p* < 0.001). Based on the multiple regression analysis, PROCESS V3.3 (Model 4) developed by Hayes was used to testify the proposed parallel mediation model. Statistics for the direct and indirect effects of perceived teacher support on students’ academic engagement were calculated. Simultaneously, bootstrap sampling using 5,000 interactions with a confidence interval of 95% was adopted to testify the significance of the mediation model (see Table 5).

**TABLE 4:** Regression analysis results (*N* = 1094).

Regression equations		Fit index			Coefficient			Collinearity statistics	
			
Predictor	Outcome	*R*	*R* ^2^	*F*	β	*B*	*t*	Tolerance	VIF
Teacher support	Engagement	0.491	0.241	347.7***	0.491	1.383	18.6		
Teacher support	Enjoyment	0.613	0.376	657.1***	0.613	0.763	25.6		
Teacher support	Boredom	0.424	0.180	239.3***	–0.424	–0.172	–15.5		
Teacher support	Engagement	0.581	0.338	185.6***	0.237	0.669	7.5	0.576	1.737
Enjoyment					0.318	0.720	9.8	0.599	1.670
Boredom					–0.139	–0.961	–0.4.9	0.756	1.322

B represents Unstandardized Coefficients, β represents Standardized Coefficients. ***p < 0.001. Teacher support, students’ perceived teacher support; engagement, academic engagement.

**TABLE 5:** Analysis of the parallel mediation model.

Pathway	Indirect effect size	SE	BCa 95% CI	Indirect/total effect (%)
Total indirect effect	0.254	0.033	[0.171,0.341]	51.63%
Perceived teacher support → enjoyment → academic engagement	0.195	0.038	[0.099,0.294]	39.63%
Perceived teacher support → boredom → academic engagement	0.059	0.015	[0.023,0.101]	12.00%
CI (boredom vs. enjoyment)	–0.136	0.047	[–0.256,–0.016]	
Direct effect	0.237			48.37%
Total effect	0.491			

All coefficients except CI are completely standardized coefficients, CI refers to confidence interval.

As can be seen from Table 5, 95% confidence intervals did not include zero (from 0.17 to 0.34), indicating that enjoyment and boredom collectively mediated the effect of perceived teacher support on students’ academic engagement. The total indirect effect size was 0.254 (i.e., a_1_b_1_ + a_2_b_2_), accounting for 51.63% of the total effect of perceived teacher support on students’ academic engagement. The indirect effect through boredom was 0.059 (a_1_b_1_), while that through enjoyment was 0.195 (a_2_b_2_). CI referred to the difference between the two mediating effect paths, i.e., the difference between the mediating effect size through boredom and that through enjoyment. It was –0.136 with the confidence interval of [–0.256, –0.016], indicating that the medicating effect of boredom was significantly smaller than that of enjoyment. More specifically, as shown in Table 5, the mediating effect of enjoyment accounted for 39.63% of the overall indirect effect size, which clearly contrasted with the 12.00% mediating effect of boredom. This indicated that enjoyment was a stronger mediator than boredom.

**FIGURE 1:**
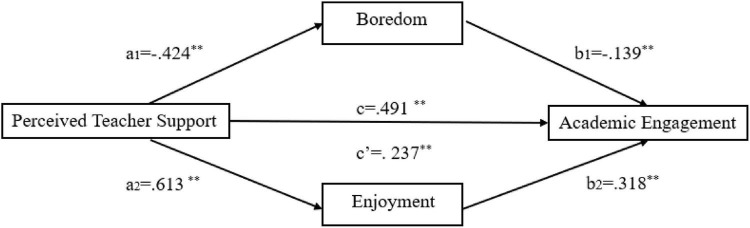
Mediating effect of enjoyment and boredom between teacher support and engagement.

As perceived teacher support could also significantly predict academic engagement (β = 0.491, *p* < 0.001), it could be concluded that perceived teacher support influenced students’ academic engagement both directly and indirectly through influencing their enjoyment and boredom. These results supported the proposed parallel mediation model (Figure 1).

## Discussion

The present study aimed to explore the relationship between perceived teacher support, enjoyment, boredom, and academic engagement. Specifically, the objectives of the present study were, first, to explore the general tendencies of students’ perceived teacher support, enjoyment, boredom, and academic engagement; second, to examine the relationship among the four variables; and finally, to determine whether enjoyment and boredom mediate the relationship between perceived teacher support and academic engagement.

With respect to the first research question, descriptive analyses indicated a general tendency that most of the students perceived a moderate level of teacher support, enjoyment, and engagement and a low level of boredom.

More than half of the students reported a moderate level of perceived teacher support (*M* = 4.07, *SD* = 0.59), indicating that students generally felt that they were supported by their teachers during English language learning. The overall level of perceived teacher support was close to that reported by junior high school students (Liu et al., 2021) and higher than that reported by university students (Sadoughi and Hejazi, 2021). This indicated that younger students were likely to perceive higher levels of teacher support. The following two reasons may help to explain the higher levels of perceived teacher support reported by younger students. On the one hand, compared with university students, pre-university students tend to spend more time with their English language teachers in school, thus they are more likely to build a close relationship with their teachers and receive more support from them. On the other hand, as teenagers are still in the period of psychological weaning (Lu, 2015), teachers are likely to give them more help and assistance during the learning process.

Most of the students reported a moderate level of enjoyment (*M* = 3.66, *SD* = 0.81) in English language learning, and it was close to that reported by international students (3.82) in Dewaele and MacIntyre’s (2014) study. Previous studies indicated that Chinese students were likely to show a lower level of enjoyment than international students (MacIntyre et al., 2019). However, as the result shows, the difference between Chinese students’ enjoyment level and that of international students was rather small. A similar result was also reported in Jiang and Dewaele’s (2019) study, which found a small difference in students’ enjoyment level between the two groups. These evidences imply that further exploration is needed before making conclusive inferences on the higher level of enjoyment in international students. Additionally, the enjoyment level was similar to that reported in Liu and Wang’s (2021) study, which also investigated Chinese high school students but lower than that in Dewaele and Li’s (2021) study, which was done among university students (2021). This suggests that university students in the skill-oriented instructional context were more likely to feel enjoyable than high school students in the examination-oriented context (Jiang and Dewaele, 2019). The discrepancy between university students’ and high school students’ enjoyment levels may also be influenced by students’ age (Botes et al., 2021). As pointed out by Dewaele and MacIntyre (2014), younger learners tended to have lower levels of enjoyment than their older counterparts.

Students generally reported a low level of boredom (*M* = 2.62, *SD* = 0.96), and their boredom level was slightly lower than that of university students in Li’s (2021) study. Due to the fact that English is not as important a major in university as it is in high school and the requirements for high school students are lower than that for college students, high school students are likely to perceive higher value and control appraisal in English language learning. Shao et al. (2020a) found that students’ boredom was negatively related to their control and value appraisals. Thus, with a perception of higher levels of control and value, high school students tend to have lower levels of boredom when compared to university students. What needs to be noticed is that although most of the students had lower levels of boredom, there was still around one-third of the students who showed a moderate level of boredom. As boredom has negative effects on students’ motivation, cognitive resources, and self-regulation (Goetz and Hall, 2014), measures need to be taken to help these students mitigate their boring feeling. It is thus advocated that future studies should pay more attention to the intervention of students’ boredom along the trend of exploring its relationship with various antecedents (Vogel-Walcutt et al., 2012).

Most students showed a moderate level of academic engagement (*M* = 3.46, *SD* = 0.91). The mean scores of it were higher than that reported in Konold et al. (2017), which were conducted among American high school students. This supports the findings of previous studies (e.g., Bear et al., 2018), which found that Asian students, especially Chinese students had higher levels of academic engagement. Meanwhile, students’ engagement level was also higher than that reported in Guo (2021), which examined the engagement of Chinese university students. High school students in China spend 3 years preparing for the College Entrance Examination (Wang and Chen, 2012), and when entering college, most of them experience a period of over-relaxation and temporary loss of goals (Zhang et al., 2015). Thus, it is reasonable for high school students to have a higher level of academic engagement in English language learning. However, more than one-fifth of the students reported that they were less engaged in their English language learning. Since learning engagement is the prerequisite for students’ language learning achievement (Zhang C. et al., 2022), the reasons behind their perceived lower level of engagement need to be further explored in qualitative studies through interpretative lens to take more contextual factors into consideration. The ecological perspective, which allows researchers to explore students’ academic engagement in various tiers of contextual factors, can be adopted in future studies (Bronfenbrenner, 1979, 1993) to gain a deeper understanding of the reason why students become disengaged in English language learning.

Concerning the second research question, correlational analyses found that significant relationships existed among the four variables. Perceived teacher support was positively related to enjoyment and academic engagement and negatively related to boredom. These results indicated that students who could get sufficient and timely help from their English teachers were likely to experience more enjoyment and less boredom, and they tended to invest more time and energy in English language learning. This echoes the findings of previous studies (e.g., Roorda et al., 2011; Wang and Eccles, 2013; Sadoughi and Hejazi, 2021), which found that perceived teacher support could help students develop positive expectations, reduce negative feelings and behaviors, and concentrate on English language learning both behaviorally and cognitively. As stated by Li et al. (2018), teacher plays an exclusive role in establishing a positive foreign language learning atmosphere. In addition, a prominent finding of the present study was that teacher support could help students relieve their English language learning boredom. This adds supplementary quantitative evidence to small-scale qualitative studies which found that students’ boredom could be partially attributed to teacher-related factors (e.g., Zawodniak et al., 2021; Kruk et al., 2022b). Theoretically, the results also provide further support for the control-value theory concerning the correlational relationship between achievement emotions and their antecedents in the foreign language learning context (Shao et al., 2019, 2020a). The effect of teacher support on students’ negative achievement emotions has been largely overlooked in previous studies in the field of language learning. In the future, it would be interesting to examine whether teacher support is related to and can relieve other negative emotions.

Moreover, enjoyment was significantly correlated with academic engagement, indicating that when students are in control of their language learning and feel enjoyable, they are more likely to participate in classroom activities. This is in accord with the finding of Sadoughi and Hejazi’s (2022) study, which also found a positive relationship between enjoyment and academic engagement. Positive emotions can provide students with energy for language learning (Zhang L. J. et al., 2022). Meanwhile, it also empirically supports the control-value theory, which assumes that positive emotions have a reciprocal relationship with students’ personal cognitive resources.

Boredom showed a negative correlation with enjoyment and academic engagement with medium effect sizes. This is in accord with the findings of Dewaele and Li’s (2021) study, which also reported a negative relationship between boredom and enjoyment. The medium effect size between boredom and engagement in the present study may be influenced by the similarities and differences between the two achievement emotions. Specifically, boredom and enjoyment share the common activity-oriented feature and show a clear difference in value and activation, i.e., boredom is deactivated and negative, while enjoyment is activated and positive (Pekrun and Stephens, 2010; Li and Li, 2022). Additionally, the negative relationship between boredom and students’ academic engagement offers empirical evidence for the conceptualization of boredom as incorporating a tendency to be cognitively and behaviorally disengaged (Goetz and Hall, 2014). This indicates that as a possible sign of whether students are engaged or not, students’ boredom should receive more attention even though it is silent in nature. As boredom was also closely related to teacher support, boredom-deducing teaching strategies should be introduced to language classrooms to help students relieve the deleterious emotion (Li, 2022).

The results of mediation analysis, pertaining to the third research question, revealed that the influence of perceived teacher support on students’ academic engagement was partially mediated by students’ enjoyment and boredom. In other words, perceived teacher support can influence students’ academic engagement both directly and indirectly through impacting their achievement emotions. Similar findings also appeared in Liu et al.’s (2018) study which found that students’ perceptions of teacher support could influence their academic engagement directly or by affecting their enjoyment in math learning. In the present study, the chain effect of perceived teacher support on academic engagement through students’ enjoyment and boredom provides empirical support for the control-value theory about the antecedents and outcomes of achievement emotions (Pekrun and Stephens, 2010) in the language learning context. Specifically, perceived teacher support as an environmental antecedent can exert an influence on students’ achievement emotions, which in turn impact their academic engagement, an important outcome of achievement emotions. These findings help broaden the applicability of control-value theory to include new antecedents and be generalized to the domain of foreign language learning (Shao et al., 2019, 2020a). As teachers’ support is closely related to students’ achievement emotions and academic engagement, it is thus recommended that teachers can adopt strategies like showing interest in students’ progress, offering evaluative feedback, and using diversified teaching strategies to support their students’ academic development (Piechurska-Kuciel, 2011).

Moreover, an interesting finding of the present study was that the mediating effect of enjoyment was stronger than that of boredom, indicating that the indirect influence of perceived teacher support on students’ academic engagement mainly works through making students feel enjoyable in their English language learning rather than alleviating their boredom. Because of the silent and invisible nature of students’ boredom (Derakhshan et al., 2022b), teachers may not easily detect it as they do on students’ enjoyment, which triggers easily observable signs such as smiling, vocalic expressions, and leaning forward (Talebzadeh et al., 2020). Moreover, students who are bored may hide this emotion to avoid potential criticism or negative evaluations from their teachers (Kruk and Zawodniak, 2018). The above-mentioned two aspects may partly explain why teachers’ further influence on students’ engagement through influencing their boredom was weakened. The finding is consistent with Liu et al.’s (2021) study which indicated that although support from the teacher can significantly enhance students’ positive achievement emotions in the classroom, it can only act as a buffer against negative achievement emotions. As boredom may make students pursue alternative goals (Bench and Lench, 2013), bored students are likely to disengage from what is going on in the classroom and neglect the teachers’ behaviors and discourse, and thus cannot benefit from teachers’ support. However, this does not mean that teacher-related variables are not important for students’ boredom. Previous studies (e.g., Dewaele and Li, 2021; Li, 2022) confirmed that teachers’ enthusiasm and teaching styles could significantly influence students’ boredom. Thus, future studies should be conducted to explore what teacher-related factors influence students’ boredom the most and find out effective ways to reduce this negative emotion.

The influence of perceived teacher support and students’ achievement emotions on students’ academic engagement indicated that both contextual factors and students’ achievement emotions are important for students’ academic engagement. The findings are consistent with Dewaele and Li’s (2021) study, which also highlights the influence of teacher enthusiasm and emotional factors on students’ social-behavioral learning engagement. Moreover, a recent study from the ecological perspective on students’ engagement in writing feedback found that alignment between context and the learner played a central role in students’ engagement in writing feedback (Han, 2019), thus future studies on students’ academic engagement should also pay attention to the interaction between learners’ emotional factors and teacher-related factors in various tiers of the system to have a more comprehensive understanding of how these factors influenced students’ engagement.

## Limitations and future research suggestions

The present study also has some limitations. First, data used in the present study were merely collected through self-report scales, which means that participants’ responses may be influenced by social desirability bias. Taking engagement as an example, future studies can adopt other measures to capture engagement in real-time and conduct data triangulation. For example, Lambert et al. (2016) measured students’ engagement using diversified and context-specific indicators such as the number of words produced and the amount of time invested. Second, this study only adopted the quantitative method, which makes it difficult to reveal the complexity caused by students’ individual differences. In the future, in order to provide a deep understanding of the influence of teacher support on students’ achievement emotions and academic engagement, data can be collected through multiple sources, including classroom observation and interviews. Third, boredom was only measured by three items. In the future, studies can adopt scales with more items to evaluate students’ boredom. Fourthly, as the questionnaires were distributed to 21 classes, the multi-level structure of the data was ignored. Future studies can adopt doubly latent multilevel analysis to explore these relationships both at the individual and class levels (Shao and Parkinson, 2021). Finally, no covariates were controlled in the present study. As gender and age may also influence students’ academic engagement (e.g., Martin-Storey et al., 2021), future studies need to take these covariates into consideration.

## Conclusion and implications

The present study explored the relationship between perceived teacher support, boredom, enjoyment, and academic engagement and testified the mediating role of boredom and enjoyment in the relationship between perceived teacher support and academic engagement. The results showed that Chinese high school students perceived moderate levels of perceived teacher support, enjoyment, and academic engagement. It also revealed that perceived teacher support was closely related to students’ enjoyment, boredom, and academic engagement. Additionally, boredom and enjoyment played mediating roles in the relationship between perceived teacher support and academic engagement. These findings not only identified the critical role of teacher support in students’ achievement emotions and academic engagement but also provided supporting evidence for the importance of students’ achievement emotions in influencing students’ academic engagement, thus highlighting the necessity of studying students’ achievement emotions in foreign language learning.

This study has important theoretical as well as practical implications. Theoretically, the high correlation between enjoyment and engagement offers further supporting evidence for the assumption that enjoyment is an important indicator of engagement, especially its emotional dimension. Meanwhile, the positive relationship between enjoyment and engagement, and the negative relationship between boredom and engagement also provide empirical evidence for the broaden-and-build theory of achievement emotions in the foreign language learning context. Practically, the findings of the present study provide supporting evidence for the effects of teacher support in helping students generate language learning enjoyment, reduce language learning boredom and become more engaged in language learning. Teachers can take some efforts to make students feel support from them. For example, they can emotionally connect with their students, encourage them to ask questions, and treat them equally (Tennant et al., 2015). This result also provides valuable implications for the design of teacher development programs. These programs should include sections to help teachers learn how to provide effective support for their students from emotional, instrumental, appraisal, and informational aspects. Additionally, given the important role of students’ emotions on their academic engagement, these programs should also increase teachers’ awareness of the importance of students’ achievement emotions and help them learn how to regulate students’ emotions more effectively.

## Data availability statement

The raw data supporting the conclusions of this article will be made available by the authors, without undue reservation.

## Ethics statement

Ethical review and approval was not required for the study on human participants in accordance with the local legislation and institutional requirements. Written informed consent to participate in this study was provided by the participants’ legal guardian/next of kin.

## Author contributions

YZ: conceptualization, validation, formal analysis, methodology, investigation, and writing-original draft. LY: writing-review and editing, resources, project administration, and funding acquisition. Both authors contributed to the article and approved the submitted version.
